# The influence of ABCB1 rs4148738 (C>T) polymorphism on rivaroxaban exposure and bleeding risk in Iraqi patients with non-valvular atrial fibrillation

**DOI:** 10.5339/qmj.2026.10

**Published:** 2026-03-17

**Authors:** Haider Khudhair Jalel, Mazin Ouda Hamid, Mohammed Hamzah Ibadi

**Affiliations:** 1 Department of Pharmacology and Toxicology, College of Pharmacy, University of Al Ayen Iraqi, Thi-Qar, Iraq; 2 Department of Pharmacology and Toxicology, College of Pharmacy, University of Karbala, Karbala, Iraq; 3 Department of Clinical Pharmacy, College of Pharmacy, University of Ahl Al Bayt, Karbala, Iraq *Email: haiderkhudhair96@gmail.com

**Keywords:** Rivaroxaban, bleeding events, non-valvular, atrial fibrillation, ABCB1

## Abstract

**Background:**

Atrial fibrillation (AF) is a common arrhythmia linked to thromboembolic risks. Rivaroxaban, a direct oral anticoagulant, prevents strokes in non-valvular AF (NVAF) patients. However, the role of genetics in its pharmacokinetics and outcomes remains unclear.

**Aim of study:**

To explore the link between the ABCB1 (rs4148738 C>T) polymorphism, rivaroxaban steady-state plasma levels, and the occurrence of bleeding events in AF patients.

Patients and methods: This cross-sectional study examines patients with AF treated with rivaroxaban anticoagulation from September 2024 to March 2025. We gathered clinical data covering demographics, comorbidities, and treatment adherence. Biochemical tests assessed renal function (serum creatinine and urea), and the steady-state plasma concentrations of rivaroxaban were determined via high-performance liquid chromatography. Genotyping was conducted using allele-specific polymerase chain reaction.

**Results:**

The study enrolled 100 participants (45 males, 55 females), most aged >46 years, with an obesity prevalence of 52%. The genotype distribution was noted as 26% CC, 39% CT, and 35% TT. Plasma levels of rivaroxaban were significantly lower in homozygous mutants (TT) compared to wild-type genotypes (*P* = 0.001). The rs4148738 polymorphism was associated with bleeding events, all of which occurred in CT carriers. This observation should be considered preliminary due to the limited sample size.

**Conclusion:**

The ABCB1 rs4148738 polymorphism influences rivaroxaban plasma levels in NVAF patients and shows a potential link to bleeding risk.

## 1. INTRODUCTION

Atrial fibrillation (AF) is classified as valvular or non-valvular according to recent ESC guidelines. Non-valvular AF (NVAF) is an irregular heart rhythm not linked to major valve disease, such as mitral stenosis or mechanical heart valves.^1^ AF is a prevalent, persistent arrhythmia, affecting approximately 50 million individuals worldwide, representing 40% of all heart rhythm disorders.^2^ The Global Burden of Diseases, Injuries, and Risk Factors Study (GBD) illustrates that the data estimated the global population affected by AF reached 55.4 million in 2022.^3^ AF increases the risk of stroke five times, and preventing stroke has consistently been essential in the treatment of this condition. Rivaroxaban has become a preferred alternative to warfarin as an oral anticoagulant due to warfarin’s narrow therapeutic index, interactions with food and drugs affecting cytochrome P450 (CYP) isoenzyme pathways, the necessity for frequent laboratory testing, and its heightened bleeding risk profile.^4^ Despite the lack of routine coagulation monitoring for direct oral anticoagulants (DOACs), the incidence of severe bleeding episodes has occasionally been documented in individuals using these medications. Rivaroxaban ranked among the top ten pharmaceuticals linked to emergency department visits due to adverse medication events in the United States during 2013 to 2014.^5^ The most common adverse events associated with rivaroxaban were hemorrhagic complications, particularly gastrointestinal bleeding, epistaxis, genitourinary bleeding, and bleeding from skin or wound sites. According to in vitro and in vivo drug interaction studies, P-glycoprotein (P-gp, ABCB1) is the carrier responsible for the active renal secretion of rivaroxaban.^6^ Genetic variations among individuals in drug-metabolizing enzymes and transporters influence the effectiveness and safety of various medications.^7^ Multiple genetic factors account for 20% to 95% of the diversity in medication responses among individuals.^8^ The ABCB1 gene encodes P-gp, a key efflux transporter expressed in barrier and excretory tissues. By limiting intestinal absorption and facilitating renal and hepatic clearance, P-gp reduces systemic drug exposure and contributes to first-pass elimination of orally administered medications.^9^ Genetic variability is a recognized determinant of rivaroxaban pharmacokinetics and safety. Wang et al. examined AF patients of Mongolian descent and found that rs1128503, but not rs4148738, influenced trough concentrations, and there was no association with bleeding.^10^ Wu et al. reported in a prospective multicenter study of NVAF patients that rs4148738 and rs4728709 significantly reduced dose-adjusted trough concentrations, though bleeding risk did not differ across genotypes.^11^ Cosmi et al. extended this evidence to a broader cohort of patients receiving DOACs, demonstrating that rs4148738 carriers had lower peak rivaroxaban levels, but bleeding outcomes were not assessed.^12^ More recently, Mardi et al. conducted a systematic review and meta-analysis including 4721 participants, which identified ABCB1 rs1045642 as a significant predictor of rivaroxaban concentrations, with carriers of the C allele showing higher plasma levels, although pooled analyses did not confirm a direct association with adverse drug reactions.^13^

The use of DOACs has increased significantly over the past two years. Nevertheless, there is a lack of studies examining their pharmacogenomics. Accordingly, the present study investigates rs4148738 in Iraqi NVAF patients to clarify its impact on rivaroxaban exposure and bleeding risk.

## 2. MATERIALS, PATIENTS, AND METHODS

### 2.1 Study design

Between September 2024 and March 2025, we evaluated the rs4148738 single-nucleotide polymorphism (SNP) in 100 NVAF patients on 20 mg rivaroxaban daily for at least 6 months. Clinical data (sex, height, weight, renal function, drug levels) and bleeding events were recorded. Inclusion criteria: age > 18 years, confirmed NVAF, and informed consent. Exclusion: valvular AF, high bleeding risk, recent or hemorrhagic stroke, liver disease, pregnancy/lactation, drug interactions (CYP3A4/P-gp inhibitors/inducers), hypersensitivity, and contraindications. Ultimately, we enrolled 100 patients with AF: 45 men and 55 women. The study was approved by the University of Kerbala Ethics Committee (IRB approval no.: 2189-6; dated: September 25, 2024), and informed consent was obtained from all participants.

### 2.2 Collection of samples and measurement of rivaroxaban trough plasma levels

After reaching steady-state (~5 half-lives), blood samples were collected for trough-level analysis and stored at −20 °C. Plasma rivaroxaban levels were measured using a validated high-performance liquid chromatography method (SYKAM, Germany) with a C18-ODS column (250 × 4.6 mm).^14^ Conditions: 40 °C column temp, 1.2 mL/min flow rate, isocratic elution (ACN:Water 55:45 v/v), 100 μL injection, UV detection at 249 nm. Peaks confirmed by retention time.

### 2.3 Genotyping of the single-nucleotide polymorphisms

The current study focuses on the ABCB1 gene (rs4148738). Following DNA extraction, allele-specific polymerase chain reactions (PCRs) were performed. PCR products were subsequently analyzed by gel electrophoresis using a UV transilluminator.

### 2.4 Primers

The primers were created by Professor Dr. Hassan Mahmood utilizing Primer Blast software (https://www.ncbi.nlm.nih.gov/tools/primer-blast/) and sent to Macrogen for additional manufacture, shown in Table 1. PCR conditions were optimized to ensure reliability and reproducibility within the amplification program.

### 2.5 Statistical analysis

Patient data were entered from clinical sheets and analyzed using SPSS v26. Results were summarized as frequencies, percentages, means, or medians (IQR) in relevant tables. For normally distributed data, ANOVA with post hoc analysis was applied; for non-normal data, Kruskal-Wallis with Bonferroni correction was used. Chi-square tested genotype distribution against the Hardy–Weinberg equilibrium and its association with bleeding events. Multiple linear regression evaluated the genetic impact on study parameters. Statistical significance was set at *P* ≤ 0.05.

## 3. RESULTS

A total of 100 patients were enrolled, comprising 45 males and 55 females. Each patient was administered rivaroxaban at a dose of 20 mg once daily, and the mean duration of treatment was 27.6 months. The demographic characteristics of these patients are detailed in Table 2. Results regarding the distribution of the ABCB1 genotype can be found in Table 3. Notably, the allele frequency distribution of this gene shows a deviation from the Hardy–Weinberg equilibrium.

**Figure 1:**
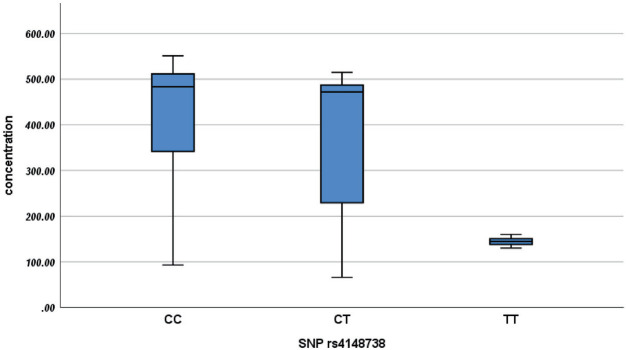
Boxplot of rivaroxaban concentrations by genotype (CC, CT, TT).

As shown in Table 4 and Figure 1, a significant difference in concentration is evident between wild CC (483.20 ± 175.83) and homozygous TT (144.60 ± 14.10), as well as heterozygous CT (471.90 ± 317.60), when compared to the homozygous TT (144.60 ± 14.10) concentration, with a P-value of 0.001 for each group. The concentrations in the homozygous TT genotype are considerably lower than those in the wild CC and heterozygous CT genotypes. These findings suggest a pronounced genetic influence on rivaroxaban plasma concentrations.

We use a multiple linear regression model to predict concentration levels using the demographic and genetic variables shown in Table 5. The model demonstrates overall statistical significance (*P* < 0.001), suggesting that the predictor variables collectively exhibit a significant relationship with concentration. In this model, age and body mass index are not significant predictors of concentration levels. Genetic as SNPs, exhibit substantial and statistically significant effects. Individuals with the mutant homozygous TT genotype exhibit a pronounced reduction of approximately 268 units compared to those with the wild CC genotype. The findings indicate that SNP genotype significantly affects concentration levels, potentially through gene expression changes and rivaroxaban transport.

**Figure 2:**
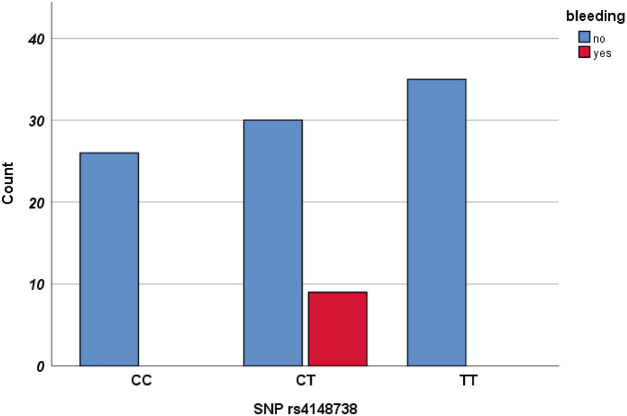
Clustered bar chart of bleeding events by genotype (CC, CT, TT).

Hemorrhagic events were systematically evaluated among the enrolled participants. Out of 100 patients, nine experienced such events, with no fatal hemorrhages reported. The most frequent types of bleeding included epistaxis and gum bleeding. We examined potential correlations between hemorrhagic incidents and polymorphisms in the ABCB1 gene. A significant association was found between variants at the rs4148738 locus and the incidence of bleeding events, particularly in the heterozygous CT, showing a strong significant association (*P* < 0.001). The findings are detailed in Table 6 and Figure 2.

## 4. DISCUSSION

Interindividual variability in response to the medication rivaroxaban may be partly attributed to gene variations encoding the transporter P-gp.^15^ Nevertheless, information about the impact of genetic variants on pharmacological responses remains controversial. This cross-sectional study demonstrates an association between genetic variants and rivaroxaban steady-state trough plasma concentrations, underscoring the need for further genetic analyses. The ABCB1 gene rs4148738 (C>T) was amplified using PCR. The results demonstrated that the T allele was the predominant allele, while the C allele was the recessive allele in the population we studied, resembling the findings observed in the Chinese population.^16^ Distributions of genotypes are deviated from the Hardy–Weinberg equilibrium, indicating potential biological or population-level factors influence this locus, which may reflect selective pressures, population differences, non-random mating, or technical factors such as small sample size. This observation should be confirmed in larger, multi-center studies.

This study is the first to assess how various genetic variations influence the steady-state concentration of rivaroxaban and bleeding incidents in patients with NVAF. The concentration results from this study indicate a statistically significant difference is evident between wild CC and mutant homozygous TT, as well as mutant heterozygous CT, when compared to the mutant homozygous TT concentration, with a *P*-value of 0.001 for each group. These findings were similar to a previous study.^11^ Which reported for the first time that the ABCB1 rs4148738 gene polymorphisms significantly influence the C_trough_/D of rivaroxaban in individuals with AF. Our results indicate that variations in the P-gp produced by the ABCB1 gene may influence the plasma concentrations of rivaroxaban. In vitro studies suggest that several SNPs in the ABCB1 gene may correlate with P-gp expression and function alterations.^9^ The findings highlight the functional consequences of the ABCB1 rs4148738 polymorphism, likely through the modulation of gene expression, transporter efficacy, by reduced mRNA stability or altered splicing efficiency, potentially resulting in reduced P-gp levels. In these situations, diminished drug efflux may result in elevated intracellular accumulation and consequently greater plasma concentration. A significant correlation was found between the ABCB1 gene polymorphism and bleeding events. Several studies have shown a link between ABCB1 C>T polymorphisms and a heightened chance of bleeding. Research conducted by Mardi et al.^13^ indicated that patients with the TT genotype, linked to diminished P-gp expression, had elevated plasma levels of DOACs, contributing to an elevated probability of hemorrhage. Multiple investigations indicate there is no significant relationship between the rs4148738 C>T mutation and clinical effects. A study conducted by Cosmi et al.^12^ has demonstrated no notable differences in bleeding probability across different ABCB1 genotypes in people taking rivaroxaban. A reasonable explanation for the different outcomes observed in CT carriers is related to the role of P-gp in rivaroxaban pharmacokinetics. P-gp expression may influence rivaroxaban absorption, distribution, and excretion, owing to differing levels of dependence on this transporter. The ABCB1 C>T polymorphism can result in either elevated or reduced P-gp expression, and heterozygous carriers (CT) may exhibit intermediate or unstable transporter activity. This variability could lead to altered rivaroxaban exposure in certain individuals, thereby increasing susceptibility to bleeding. Moreover, differences in study populations, co-medication, and clinical characteristics may contribute to inconsistencies across investigations. Taken together, these findings highlight the need for cautious interpretation of the CT-bleeding association and underscore the importance of validation in larger, multicenter studies. Wu et al. demonstrated that polymorphisms in the ABCB1 gene showed no correlation with bleeding events when using rivaroxaban.^11^

## 5. LIMITATIONS OF THE STUDY

The study included 100 Iraqi patients. We suggest conducting similar studies with a larger sample size, multi-center, in specific target populations.

## 6. CONCLUSION

The findings indicate that ABCB1 rs4148738 variants are correlated with rivaroxaban plasma concentrations, and a significant correlation was found between the rs4148738 locus and bleeding events. The relationship between genetic variants and bleeding occurrences necessitates confirmation through a larger sample size.

## Figures and Tables

**Table 1.** Primer sequence of ABCB1 gene rs4148738 C>T.

Primer set tubes	Nucleotide sequence	Product length
Rs 4148738		
Forward C allele	5-TGAGGGGAGGAACTAAAACCC-3	
Forward T allele	5-TGAGGGGAGGAACTAAAACCT-3	260
Reverse common	5-AGACACCTCAAACTTGGCCC-3	

**Table 2.** Descriptive statistics of the demographic characteristics of the studied patients (*N* = 100).

Variable		*N*	Percent
Sex	Male	45	45
	Female	55	55
Age	30–45	9	9
	46–60	42	42
	>60	49	49
Body mass index	Underweight	0	0
	Healthy weight	13	13
	Over weight	35	35
	Obese	52	52
Compliance	Good	89	89
	Fair	1	1
	Bad	10	10
Duration of treatment (months)	6–42	85	85
	>42	15	15
Diabetes mellitus	Yes	36	36
	No	64	64
Hypertension	Yes	85	85
	No	15	15
Bleeding event	Yes	9	9
	No	91	91

**Table 3.** Distribution of gene polymorphism for SNP rs4148738 genotype in patients (*N* = 100).

Variable		Frequency	Percent
Genotype	CC wild	26	26
SNP rs4148738	CT hetero	39	39
	TT homo	35	35

**Table 4.** Association of renal function parameters and concentration level with different genotypes of the ABCB1 gene C>T (rs4148738) polymorphism in patients.

Parameter	CC	CT	TT	MC	*P* value
S.Cr.	0.800 (0.3)	0.900 (0.2)	0.900 (0.3)	NS	0.431
Conc.	483.20 (175.83)	471.90 (317.60)	144.60 (14.10)	CC vs. TT	0.001*
				CT vs. TT	0.001*
B.U.	38.36 ± 10.94	37.62 ± 9.43	38.20 ± 5.95	NS	0.847

CC, CT, TT: Genotypes; S.Cr.: Serum creatinine; Conc: Plasma concentration; B.U.: Blood urea. *P* values less than 0.05 were considered statistically significant. Bold values with asterisks indicate statistically significant differences between genotypes.

**Table 5.** Multiple linear regression model predicting concentration levels based on demographic and genetic variables for ABCB1 rs4148738 genotypes.

*R*	*R* ^2^	Adjusted *R*^2^	SE of the estimate	Model *P* value
0.700	0.490	0.469	125.165	<0.001*
**Predictor variable**		**Unstandardized coefficients**		***P* value**
		***B***	***SE***	
Age		0.569	1.267	0.654
BMI		0.821	2.120	0.699
CT – CC (Ref)		−27.954	31.711	0.380
TT – CC (Ref)		−267.998	32.615	<0.001*

*P* values less than 0.05 were considered statistically significant. Bold values with asterisks indicate statistically significant associations between genotype and bleeding events.

**Table 6.** Association of genetic variation and bleeding events.

SNP	Genotype	Bleeding	No-bleeding	*P* value
rs4148738	CC	0	26	<0.001*
	CT	9	30	
	TT	0	35	

*P* values less than 0.05 were considered statistically significant. Bold values with asterisks indicate statiestically significant associations between genotype and bleeding events.

## Data Availability

Supporting data are available from the corresponding author upon reasonable request. Due to confidentiality, individual patient data are restricted, but anonymized data may be shared per ethical guidelines.
